# Bibliographical

**Published:** 1892-02

**Authors:** 


### Bibliographical—A New Journal.

*The Bacteriological World and Modern Medicine*—Bulletin of the Laboratory of Hygiene Sanitarium, edited by Paul Paquin, M.D., D.V.S.

The first number of the first volume of this journal is very presentable indeed; it is to be devoted, as we understand it, to the development, both theoretical and practical, of the subject of bacteriology as well as modern medicine.

This, however, is the successor of a journal that was published during 1890. One of the editors, Dr. J. H. Kellogg, in speaking of the purpose of this journal, says: "Probably nothing has contributed more to the advancement of medical science in the last quarter of a century than the growing tendency to specialism, and the development of various specialties in medical practice. The rapid accumulation of important and even revolutionary facts in the various departments of medical knowledge, especially within the last twenty-five years, has made it impossible for any one mind to grasp the whole of medical science, and has rendered it necessary that one who would become in the highest degree proficient as a surgeon or medical practitioner should devote himself to one particular line of study and research as the only means of attaining the desired end. While it may be conceded that the tendency to specialism has been carried somewhat too far, the great advantages which have resulted from the labors of the specialist must be regarded as one of the most important elements of progress in modern medicine. * *

"It will be the aim of the editors of this journal to make the pages an epitome of the fullest and most advanced thought and the ripest experience upon all subjects which come within its scope. *The Bacteriological World and Modern Medicine* is not designed for a special class."

The known ability of the editors of this journal is a sufficient guarantee that the purpose they have outlined will be carried out to the fullest, which will make this journal one of inestimable value to men in all departments and specialties of medical science and practice.

